# Topical *Bambusa vulgaris* Extract Enhances Wound Healing in Cutaneous Leishmaniasis

**DOI:** 10.1155/2021/7860474

**Published:** 2021-02-11

**Authors:** Zahra Ghanbarinasab, Mahnaz Hosseini-Bensenjan, Elaheh Ziaei Ziabari, Shiva Aminnia, Roham Borazjani, Mohammad Rastegarian Jahromi, Qasem Asgari, Bahador Sarkari, Soheil Ashkani-Esfahani

**Affiliations:** ^1^Student Research Committee, Shiraz University of Medical Sciences, Shiraz, Iran; ^2^The Rothman Institute, Department of Orthopaedic Surgery, Thomas Jefferson University, Philadelphia, Pennsylvania, USA; ^3^Trauma Research Center, Shahid Rajaee (Emtiaz) Trauma Hospital, Shiraz University of Medical Sciences, Shiraz, Iran; ^4^Department of Parasitology and Mycology, School of Medicine, Shiraz University of Medical Sciences, Shiraz, Iran; ^5^Basic Sciences in Infectious Diseases Research Center, Shiraz University of Medical Sciences, Shiraz, Iran

## Abstract

**Background:**

*Bambusa vulgaris* (Tabashir) has been shown to have antimicrobial, antioxidant, and anti-inflammatory effects due to the presence of ascorbic acid, vitamin B2, flavonoid, and phenolic compounds which can be beneficial in the process of wound healing. The current study aimed to evaluate the effects of topical Tabashir extract on cutaneous leishmaniasis (CL) caused by *Leishmania major* in BALB/c mice.

**Methods:**

Twenty-eight female BALB/c mice (4 weeks old, 18 ± 4 grams) were injected subcutaneously in tail-base with *L. major* amastigotes. Treatment started when the CL lesions were appeared and continued for 21 days. Mice were then divided into four groups: *E*1, treated daily with 5% of Tabashir extract gel; *E*2, treated daily with 10% Tabashir gel; *C*1, irrigated daily only with normal saline; and *C*2, received vehicle gel daily. The wounds' sizes were measured every 3 days, using vernier caliper. The volume densities of vessels, collagens, and hair follicles, vessels' length density, and mean diameter were soteriologically determined.

**Results:**

Tabashir enhanced wound closure rate through increasing the number of fibroblasts, collagen bundles, and vessels, according to histomorphometric evaluation while it did not affect the parasitic load. Findings of the in vitro study revealed that the extract has substantial mortality for the *Leishmania* promastigotes.

**Conclusion:**

Topical Tabashir showed promising effects on the healing process of skin wounds caused by CL in this experimental study. Further studies are suggested to find out the molecules which are involved in the healing process.

## 1. Introduction

Leishmaniasis is a global issue that annually affects 600,000–1,000,000 new cases worldwide and is caused by more than 20 species of an intracellular parasite of the genus *Leishmania* [1]. Leishmaniasis ranges from a self-limited disease with few or even no sequelae to life-threatening and destructive visceral form, generally in immunocompromised patients [2]. The most common form of the disease is cutaneous leishmaniasis (CL). According to the World Health Organization (WHO), leishmaniasis is one of the most neglected tropical diseases and the most prevalent one in tropical and subtropical areas such as the Middle-East, Africa, and the Mediterranean regions [3].

*Bambusa vulgaris* commonly called Tabashir, is typically a tropical and subtropical plant of the genus *Bambusa* which is related to the family of *Poaceae*. It has been documented that *Bambusa*'s leaves contain phosphorus, vitamin B2, beta-carotene, iron, flavonoids, ascorbic acid, and phenolics [4]. It has also been reported that Tabashir possesses anti-inflammatory, antimicrobial, and antioxidant activities [5, 6]. It is also useful in reducing body temperature in fever and also for reducing blood sugar in diabetes [4, 7, 8].

The goal of CL treatment is a diminution in both the duration and severity of the disease and also in scar formation. Common anti-*Leishmania* drugs used for the treatment of CL are pentavalent antimonials and pentamidine [9]. Two globally used intralesion pentavalent antimonials are sodium stibogluconate (Pentostam) and meglumine antimoniate (Glucantime). Nowadays, these drugs have limited effectiveness, mostly due to an increase in drug-resistance. Also, their side effects and toxicities, such as nephrotoxicity, pancreatitis, hepatotoxicity, and cardiac damage, are strong barriers to long-term use [10, 11].

Considering the reported potentials of Tabashir in the literature and since finding a new treatment that can improve wound healing with minimal scar formation and side effects in CL has still remained a concern for health care providers, this study aimed to evaluate the effects of hydroalcoholic extract of Tabashir on CL induced by *L. major* in BALB/c mice.

## 2. Materials and Methods

### 2.1. Preparation of Tabashir Gel

Tabashir extract was supplied by Zardband Co. (Yasuj, Iran). For better evaluation of the efficacy of the extract, 5% and 10% concentrations of Tabashir gels were prepared by dissolving 5 and 10 g of the Tabashir extract in 2 mL of distilled water, respectively. The solutions were then transferred into the vehicle, 2% carboxymethyl-cellulose solution (2 g CMC/100 mL of distilled water), to formulate the proposed concentrations for topical uses [12]. The dosage was selected based on a pilot study which was performed to decide on the lowest dosage with the best efficacy. The CMC gel itself was administered on the CL lesions in another pilot study and revealed that this gel had no significant therapeutic effect on the CL lesions, compared to the control group.

### 2.2. Ulcer Induction and Treatments

The BALB/*c* mice were kept in a standard living situation with 12 : 12-hour dark-light cycle, 40–50% humidity, at 25 ± 3°C temperature. Free access to a standard diet and water were also provided. Twenty-eight female BALB/*c* mice (4 weeks old, 18 ± 4 g weight) were obtained from Pasteur Institute, Tehran, Iran. The infection in BALB/*c* mice was induced by using the amastigote form of *L. major* (strain reference no. MRHO/IR/75/ER). To do that, 0.1 mL of a suspension, containing 4000–5000 amastigotes (counted by a hemocytometer counting-chamber), was injected subcutaneously into the top of mice's tail-base, using insulin syringes. Lesions were appeared after about 3 weeks of inoculation. Mice were divided into four groups (*n* = 7); group *E*1 treated with the 5% concentration of Tabashir gel daily; group *E*2 received 10% gel daily; group *C*1 received normal saline every day, and group *C*2 treated by vehicle gel (2%, CMC) daily. Gel administrations were started topically from the day that open wounds were appeared and continued daily until the end of the third week. The size of the lesions was measured every three days, using vernier caliper. To do this, the length of the minor and major axes of the lesion at the base of the mouse's tail perpendicular to each other was measured and the area of the lesion was calculated in square millimeters using ellipse area formula.

At the end of the treatment, the animals were euthanized using a high dose of ether. A circular skin sample with 1 cm of the grossly healthy margin around the lesion was excised from the wound's site and used for histomorphometric assessments.

### 2.3. Flow Cytometry and the Histomorphometric Study

The effect of Tabashir on the in vitro growth of parasite was estimated before the in vivo study. Tabashir extract was dissolved in dimethyl sulfoxide (DMSO) and phosphate-buffered saline (PBS), and different concentrations of the extract were prepared. Promastigotes of the parasite were incubated with different concentrations of the extract for 2 hours at the temperature of 4°C and then were collected in Eppendorf tubes and incubated for 30 minutes with propidium iodide (50 g/ml, Sigma™, USA) and then were maintained on ice till analysis. Ethanol solution (70% v/v) was used as control. FACSCalibur flow cytometer (Becton-Dickinson™, USA) and polystyrene flow cytometry tubes (BD Falcon™, USA) were used for flow cytometry.

For the histomorphometric study, nine 1 mm^2^ pieces were cut from the skin samples, were embedded in paraffin blocks, and were cut in 15 *μ*m thickness slices and stained with Heidenhain's azan-trichrome and haematoxylin and eosin (H&E) stains. The volume densities of the collagens, vessels, and follicles of hair were determined by using the stereological point counting method [12]. Vascular length density (Lv; mm/mm^3^), mean vessels' diameter (*µ*m), and fibroblasts' population (Nv; ×10^3^ per mm^3^) were estimated as previously reported [13].

### 2.4. Statistical Analysis of the Data

Statistical analyses of the results were done by SPSS software (Version 19.0, SPSS Inc. IBM Corp., USA), using the *t-test*. Data were reported as charts, mean ± standard deviation (SD). *P* value <0.05 was considered as statistically significant.

## 3. Results

The sizes of the lesions were measured every three days, using vernier caliper, and the lesion sizes were homogeneously distributed at the initial measures in the different experimental groups, as there were no significant differences in the mean of lesion sizes, when statistically analyzed. Findings of the current study revealed that Tabashir had an insignificant impact on the load of the parasite at any concentration in contrast with a group of mice that were treated topically with 70% ethanol solution. The differences in the mean surface of the lesions at the end of treatment course (day 21th) in mice treated with 5% or 10% topical Tabashir gel (*E*1 and *E*2, respectively) were statistically insignificant in comparison with the untreated control group (*C*1) and gel-base-treated group (*C*2). Findings of the in vitro evaluation of Tabashir by incubating the promastigotes of the parasite with different concentrations of the extract and flowcytometry analysis of the treated promastigotes revealed that the extract, at 10% concentration, has a mortality rate of 86% on *Leishmania* promastigotes.

**Figure 1:**
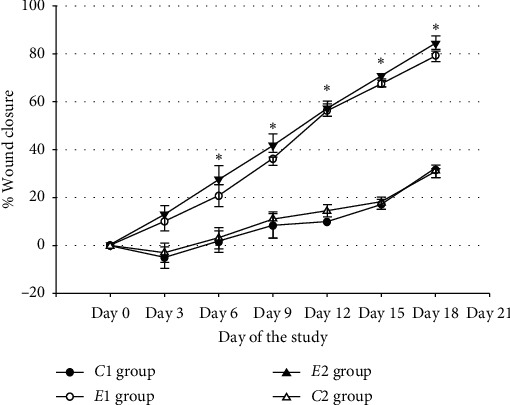
The effect of topical Tabashir on the closure rate of the wounds in BALB/c mice of the nontreated group (*C*1), gel-base-treated group (*C*2), Tabashir 5% (*E*1), and 10% (*E*2) gel-treated groups. Each point shows the mean closure percentage on a specific day; ^*∗*^a significant difference in a specific day between the *E* groups and *C* groups.

The Tabashir-treated groups have had considerably higher closure rates compared to the nontreated group (*C*1) and gel-base-treated group (*C*2), specifically from day 6 of the study (Figure 1).  Table 1 shows that Tabashir-treated groups (*E*1 and *E*2) had higher rates of collagen bundles (*P*=0.031 and *P*=0.022, respectively), vessels' volume density (*P*=0.041 and *P*=0.033, respectively), vessels' length density (*P*=0.033 and *P*=0.029, respectively), and vessels' mean diameter (*P*=0.024 and *P*=0.021, respectively), in comparison to the untreated control group. The fibroblast population (numerical density) in mice treated with 5% and 10% topical Tabashir gel (*E*1 and *E*2) was significantly higher in comparison with the untreated control group (*C*1) and gel-base-treated group (*C*2). Statistical analysis revealed no significant differences in histomorphometric parameters in *C*1 and *C*2 groups (*P* > 0.05) (Table 1).

## 4. Discussion

Leishmaniasis triggers human immunity which leads to overproduction of proinflammatory cytokines and reactive oxygen species (ROS) [13]. These changes, particularly the oxidative status of the infected site, can interfere with the process of wound healing and impair tissue regeneration [13].

Recent investigations gave more attention to natural medicines' lack of ideal drugs against Leishmaniasis. Plant derivatives are appropriate alternatives that have the potential to affect the healing process of leishmaniasis caused skin wounds [12, 14, 15]. Examples of these traditional therapies are *Peganum harmala*, *Arnebia euchroma*, silymarin, *Achillea millefolium*, *Allium sativum*, *Artemisia* species, and *Thymus vulgaris* or derivatives that have been evaluated for their antileishmanial impacts in Iran [12, 14, 15].

The effect of Tabashir on wound closure and its possible effects on CL have not been properly investigated. According to a study by Lodhi et al., Tabashir has antimicrobial properties due to the presence of flavonoid and phenolic compounds. This antimicrobial effect along with the anti-inflammatory features and the presence of antioxidants in the extract of this natural plant could be responsible for its role in collagen formation [5]. This study aimed to evaluate the efficacy of Tabashir extract in the treatment of CL. Although treatment with Tabashir in this study did not significantly decrease the parasite load, wound closure rate was significantly improved after treatment with this natural agent. Histomorphometric analysis showed that topical Tabashir gel improves collagen bundle synthesis and increases the mean of the vessel's diameter and vessels' volume density significantly which is consistent with previous evidence [5]. Unlikely, in the present study, the number of fibroblasts was not significantly increased after the administration of topical treatment.

One of the limitations of this study was not including a treatment group with common anti-*Leishmania* medications to be compared to Tabashir treatment. Another limitation was the secondary bacterial superinfection, which was not considered, that could have elongated the wound healing period and interfered with collagen formation.

Although findings of the in vitro study revealed that the extract has substantial mortality for the *Leishmania* promastigotes, however this might not be the case for the amastigotes of the parasite which is present in the human lesions. This reaffirms the findings of previous studies which showed that the results of in vitro experiments on *Leishmania* promastigotes could not be easily extrapolated to the amastigote form of the parasite.

## 5. Conclusion

The present survey showed that the topical gel of hydroalcoholic extract of Tabashir has promising effects on the healing process of skin wounds caused by CL in BALB/*c* mice models. This work is preliminary, and further study, with larger sample size, is needed to explore the mechanism of action and also to determine the active anti-*Leishmania* ingredients in this extract. Whether the compounds in this extract activate inflammatory reactions and trigger the production of proinflammatory cytokines and ROS or invoke immune system cells at the site of the lesion is the issue that needs further investigation in future studies.

## Figures and Tables

**Table 1:** Histomorphometric and stereological study outcome from full-thickness skin specimens of the leishmaniasis induced skin wounds of mice treated with topical Tabashir gel 5% (*E*1) and 10% (*E*2) in contrast with the untreated control group (*C*1) and gel-base-treated group (*C*2).

Groups	Fibroblasts	Collagen bundles	Vessels			Hair follicles
	Numerical density	Volume density	Volume density	Length density	Mean diameter	Volume density
*C*1	149.4 (11.6)	56.4% (5.7%)	1.1% (0.5%)	11.2 (9.9)	21.3 (11.4)	2.6% (2.5%)
*C*2	152.5 (12.5)	60.6% (7.9%)	1.2% (0.9%)	13.1 (9.6)	22.9 (13.7)	3.1% (1.4%)
*E*1	201.1 (42.4)	86.7% (1.4%)^∗^	3.9% (0.7%)^∗^	19.2 (8.1)^*∗*^	39.8 (19.1)^*∗*^	2.0% (0.2%)
*E*2	205.1 (87.4)^∗^	89.6% (3.4%)^∗^	3.3% (0.4%)^∗^	20.5 (11.7)^*∗*^	47.9 (16.6)^*∗*^	1.9% (1.1%)

^*∗*^ *P* value <0.05, experimental groups vs. *C*1 and *C*2.

## Data Availability

The raw data used to support the findings of this study are available on request.
